# Re: An unanswered question in pediatric urology: the post pubertal persistence of prepubertal congenital penile curvature correction by tunical plication

**DOI:** 10.1590/S1677-5538.IBJU.2017.0055.1

**Published:** 2018

**Authors:** Daniel Yachia

**Affiliations:** 1 Department of Urology, Hillel Yaffe Medical Center in Hadera, Affiliated to the B. Rappaport School of Medicine of the Technion Israel Institute of Technology, Haifa, Israel

*To the editor*,

I read with great interest the article “An unanswered question in pediatric urology: the post pubertal persistence of prepubertal congenital penile curvature correction by tunical placation” by Ozkuvanci et al. (1), where the authors conclude that “…. recurrence might be observed in half of the patients after puberty”. Table- 1 in the article shows that only 5 of the 13 patients had straight erections in adulthood and all the rest had recurrent curvatures from 10° to 50°. A recurrence rate of almost 60% is very high for any correctional surgery technique.

The etiology of congenital penile curvatures is the development differences in parts of the tunica albuginea of the corpora cavernosa. Darewitz et al. (reference 13 in the article) already described this in 2001 (2). The article claim that “It is not certain whether this is a problem related to the technique or the nature of an ongoing developmental process” is a bit misleading. Recurrence of the curvature is always because of the technique used for its correction, the experience of the surgeon and the suture material used. The human penis develops together with all other organs of the body. To the best of my knowledge, there is no separate development activities in the penile tissues. Most of the pediatric curvatures are congenital and related to the disproportional development of some parts of the t. albuginea. A few, like the “Penile chordee without hypospadias” are caused by real chordee tissue at the ventral part of the penis. Development of this chordee tissue is also congenital and do not continue developing during childhood or adulthood.

I would like also to correct a common mistake done by many authors: Neither the excisional (i.e. Nesbit, Kelami) or incisional (i.e. Yachia) corporoplasty techniques are “plicational”. The techniques used for correcting the penile curvature should be named according the manipulation. The ones based on shortening the longer part of the corpora are “shortening” procedures. The “plicational corporoplasty” of Ebbehoj and Metz (reference 2 in the article) is also a shortening repair (3). The main difference of the last one is that there are no cut edges to heal and maintain the repair. Everything depends on the non-absorbable sutures holding the plication.

The “Incisional Corporoplasty” was developed after I saw several recurrent cases of congenital penile curvatures and residual ventral curvatures after chordee excision in hypospadias cases, which were corrected by plication sutures (Yachia D. (4)). The results brought by the authors in the article are a nice proof of my claims that the plicational corporoplasty techniques have a high risk of recurrence (5). These recurrences are because of the lack of a dependable scar tissue development between the loops of the monofilament suture material to maintain the repair the deformation in a permanent manner. Children also have erections but they are not as hard as the ones in post-pubertal period, and these harder erections are the cause of a gradual “cheese-wire” cutting of the tissues by the sutures, and gradual reappearance of the curvature. I would not call them “post pubertal persistence” but “recurrence”, because I am almost sure that at the end of their pre pubertal repair the penis was completely straight in artificial erection.

Although the plicational technique is much simpler to perform, unfortunately its recurrence rates are high. When doing a corporoplasty it is worth to spend a bit more time and to use one of the excisional (recommended in adult cases) or incisional (recommended also in pediatric cases) techniques. The cut edges will perfectly heal and adhere, especially when delayed absorption sutures are used that holds their approximation tension for a month and longer, and then disappear without leaving any knots under the skin. I would also like to recommend the use of “artificial straightening” (as described by Kelami which I always use) (6) by applying Allis clamps to the apex of the convex part to decide the site and extent of the incision or excision. In most cases this will allow to do a single incision to straighten the curvature.
